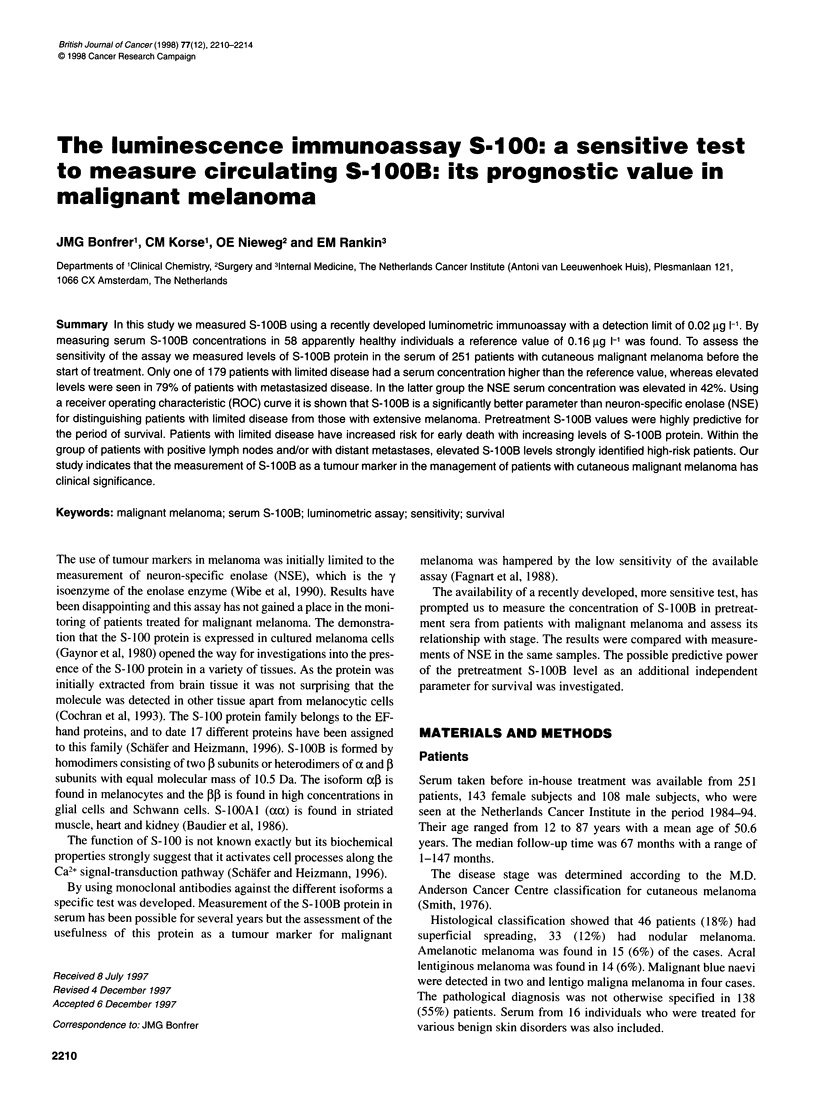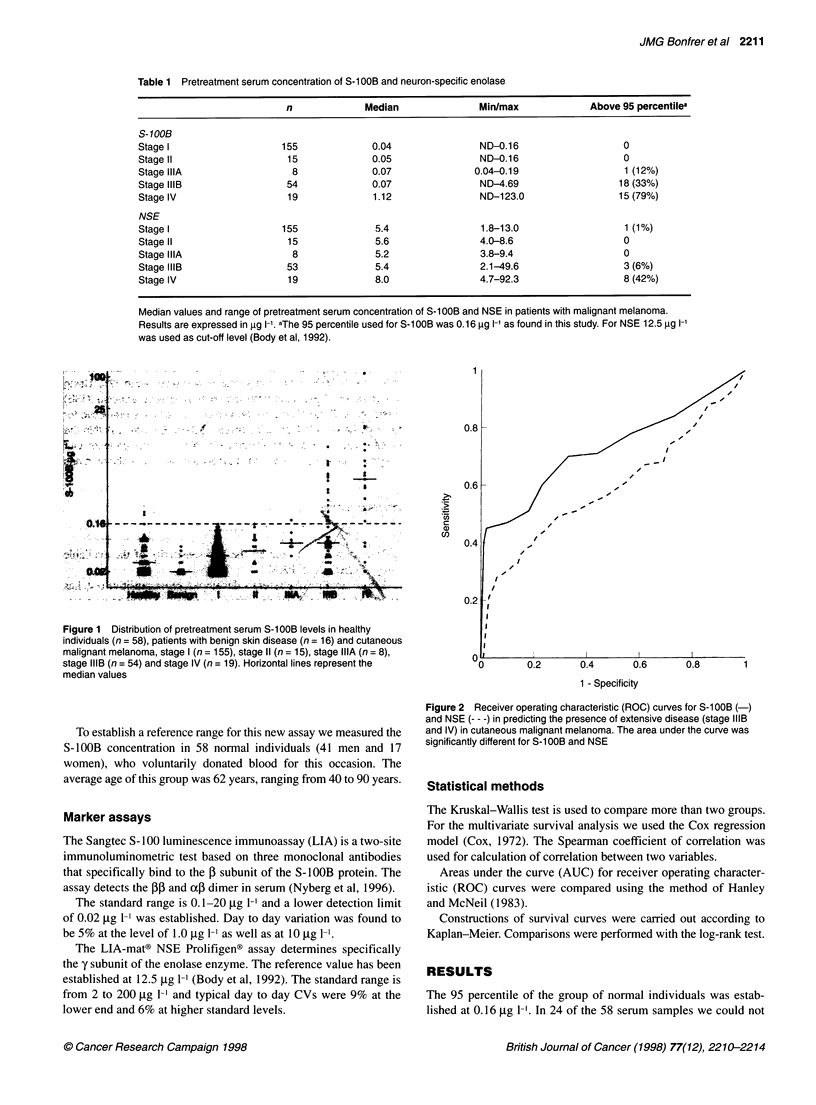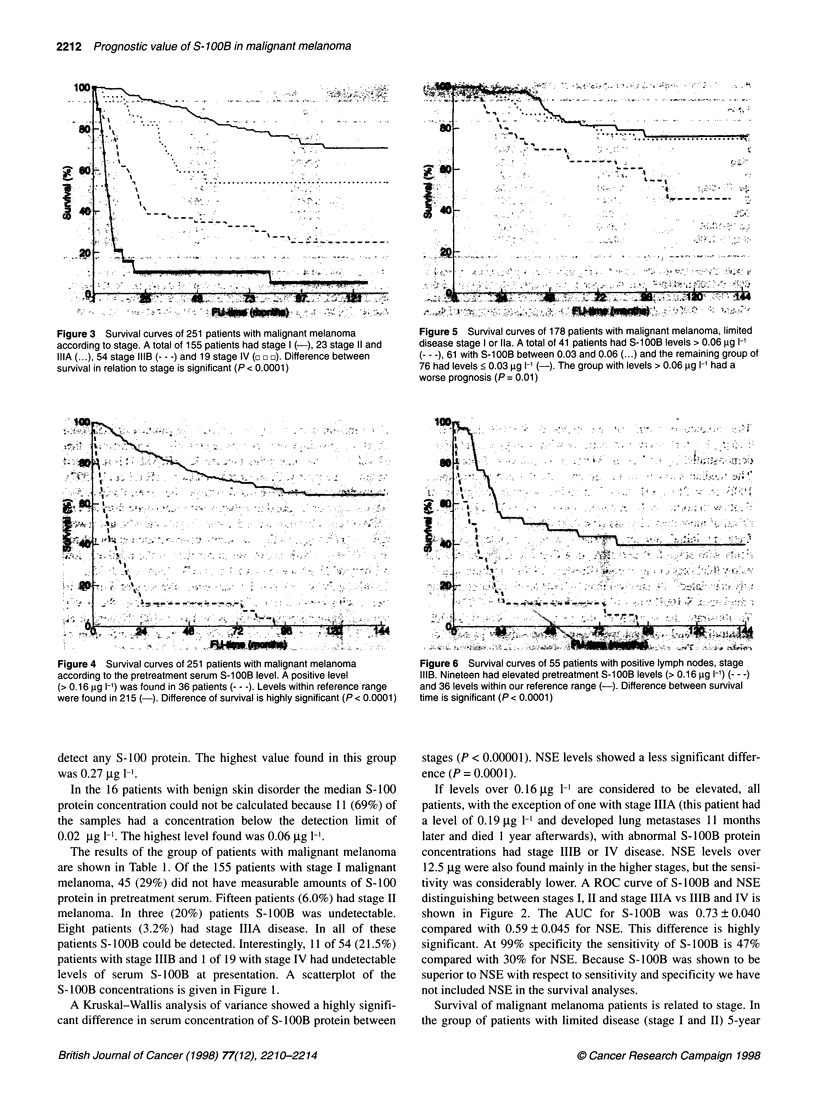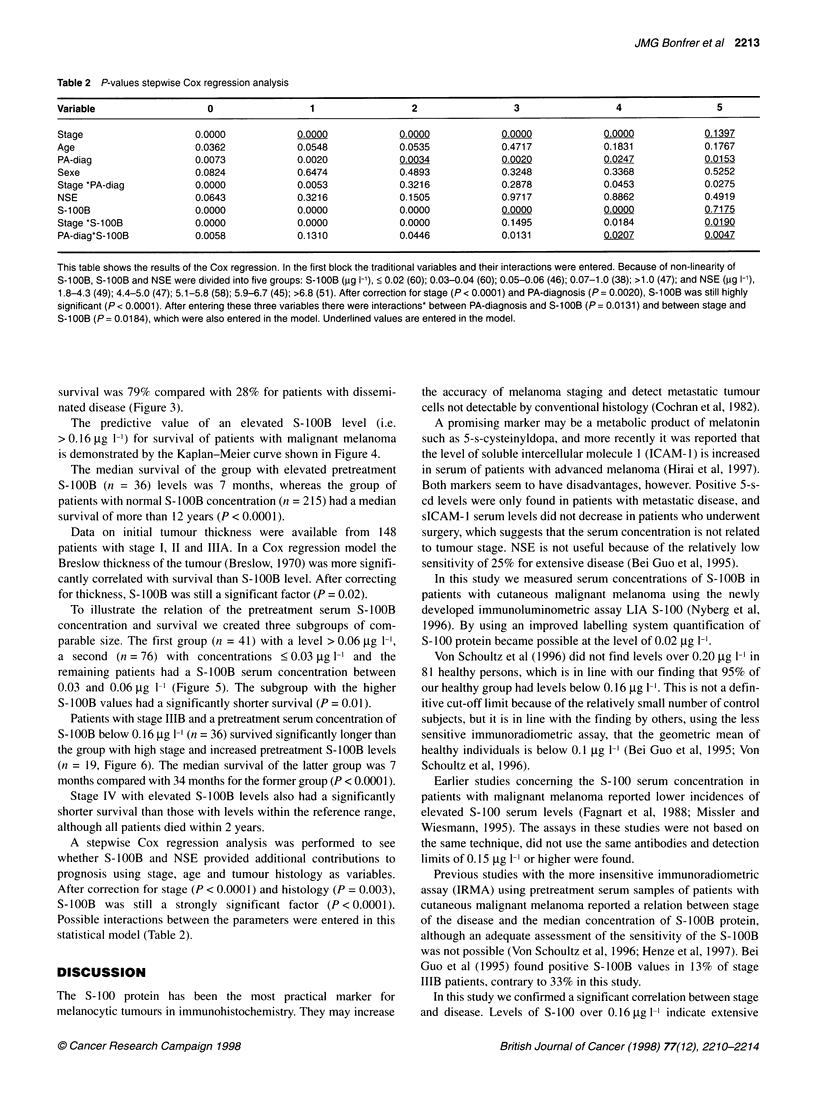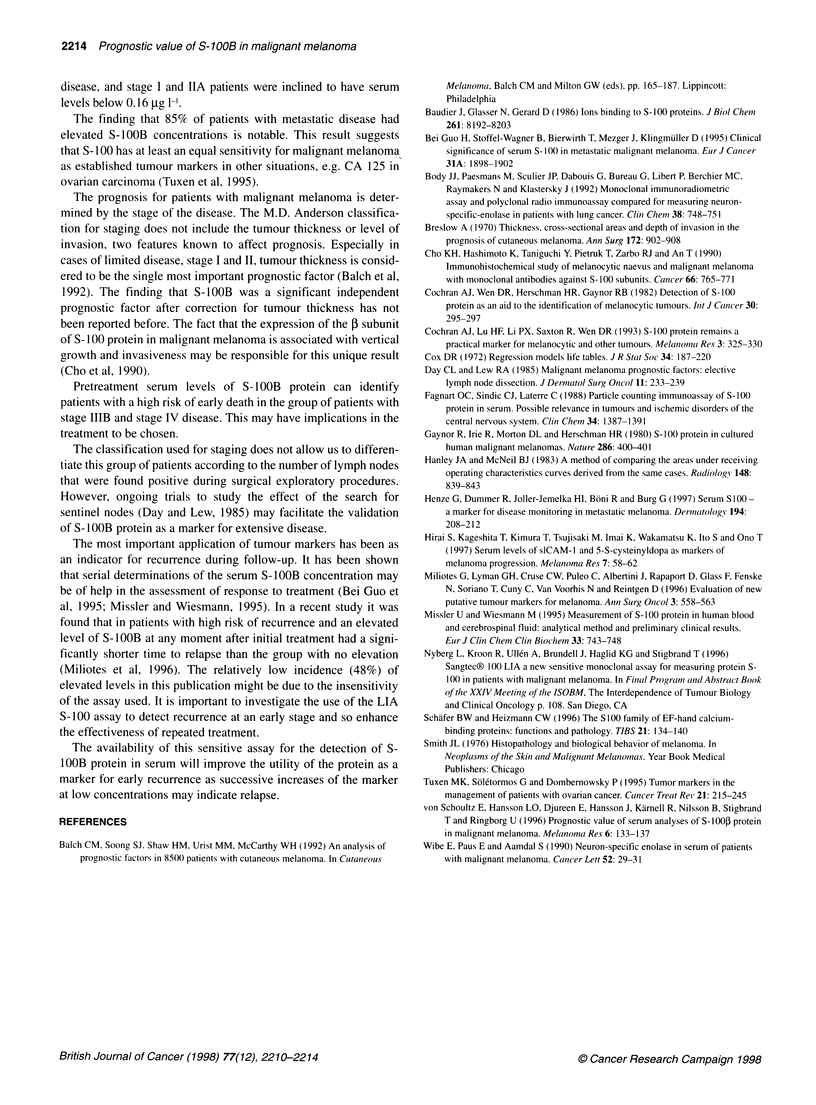# The luminescence immunoassay S-100: a sensitive test to measure circulating S-100B: its prognostic value in malignant melanoma.

**DOI:** 10.1038/bjc.1998.368

**Published:** 1998-06

**Authors:** J. M. Bonfrer, C. M. Korse, O. E. Nieweg, E. M. Rankin

**Affiliations:** Department of Clinical Chemistry, The Netherlands Cancer Institute (Antoni van Leeuwenhoek Huis), Amsterdam.

## Abstract

In this study we measured S-100B using a recently developed luminometric immunoassay with a detection limit of 0.02 microg l(-1). By measuring serum S-100B concentrations in 58 apparently healthy individuals a reference value of 0.16 microg l(-1) was found. To assess the sensitivity of the assay we measured levels of S-100B protein in the serum of 251 patients with cutaneous malignant melanoma before the start of treatment. Only one of 179 patients with limited disease had a serum concentration higher than the reference value, whereas elevated levels were seen in 79% of patients with metastasized disease. In the latter group the NSE serum concentration was elevated in 42%. Using a receiver operating characteristic (ROC) curve it is shown that S-100B is a significantly better parameter than neuron-specific enolase (NSE) for distinguishing patients with limited disease from those with extensive melanoma. Pretreatment S-100B values were highly predictive for the period of survival. Patients with limited disease have increased risk for early death with increasing levels of S-100B protein. Within the group of patients with positive lymph nodes and/or with distant metastases, elevated S-100B levels strongly identified high-risk patients. Our study indicates that the measurement of S-100B as a tumour marker in the management of patients with cutaneous malignant melanoma has clinical significance.


British Journal of Cancer (1998) 77(12), 2210–2214
© 1998 Cancer Research Campaign

### The luminescence immunoassay S-100: a sensitive test to measure circulating S-100B: its prognostic value in malignant melanoma

**JMG Bonfrer[1], CM Korse[1], OE Nieweg[2] and EM Rankin[3]**

Departments of [1]Clinical Chemistry, [2]Surgery and [3]Internal Medicine, The Netherlands Cancer Institute (Antoni van Leeuwenhoek Huis), Plesmanlaan 121, 1066 CX Amsterdam, The Netherlands

**Summary** In this study we measured S-100B using a recently developed luminometric immunoassay with a detection limit of 0.02 μg $l^{-1}$. By measuring serum S-100B concentrations in 58 apparently healthy individuals a reference value of 0.16 μg $l^{-1}$ was found. To assess the sensitivity of the assay we measured levels of S-100B protein in the serum of 251 patients with cutaneous malignant melanoma before the start of treatment. Only one of 179 patients with limited disease had a serum concentration higher than the reference value, whereas elevated levels were seen in 79% of patients with metastasized disease. In the latter group the NSE serum concentration was elevated in 42%. Using a receiver operating characteristic (ROC) curve it is shown that S-100B is a significantly better parameter than neuron-specific enolase (NSE) for distinguishing patients with limited disease from those with extensive melanoma. Pretreatment S-100B values were highly predictive for the period of survival. Patients with limited disease have increased risk for early death with increasing levels of S-100B protein. Within the group of patients with positive lymph nodes and/or with distant metastases, elevated S-100B levels strongly identified high-risk patients. Our study indicates that the measurement of S-100B as a tumour marker in the management of patients with cutaneous malignant melanoma has clinical significance.

**Keywords:** malignant melanoma; serum S-100B; luminometric assay; sensitivity; survival

The use of tumour markers in melanoma was initially limited to the measurement of neuron-specific enolase (NSE), which is the γ isoenzyme of the enolase enzyme (Wibe et al, 1990). Results have been disappointing and this assay has not gained a place in the monitoring of patients treated for malignant melanoma. The demonstration that the S-100 protein is expressed in cultured melanoma cells (Gaynor et al, 1980) opened the way for investigations into the presence of the S-100 protein in a variety of tissues. As the protein was initially extracted from brain tissue it was not surprising that the molecule was detected in other tissue apart from melanocytic cells (Cochran et al, 1993). The S-100 protein family belongs to the EF-hand proteins, and to date 17 different proteins have been assigned to this family (Schäfer and Heizmann, 1996). S-100B is formed by homodimers consisting of two β subunits or heterodimers of α and β subunits with equal molecular mass of 10.5 Da. The isoform αβ is found in melanocytes and the ββ is found in high concentrations in glial cells and Schwann cells. S-100A1 (αα) is found in striated muscle, heart and kidney (Baudier et al, 1986).

The function of S-100 is not known exactly but its biochemical properties strongly suggest that it activates cell processes along the $Ca^{2+}$ signal-transduction pathway (Schäfer and Heizmann, 1996).

By using monoclonal antibodies against the different isoforms a specific test was developed. Measurement of the S-100B protein in serum has been possible for several years but the assessment of the usefulness of this protein as a tumour marker for malignant melanoma was hampered by the low sensitivity of the available assay (Fagnart et al, 1988).

The availability of a recently developed, more sensitive test, has prompted us to measure the concentration of S-100B in pretreatment sera from patients with malignant melanoma and assess its relationship with stage. The results were compared with measurements of NSE in the same samples. The possible predictive power of the pretreatment S-100B level as an additional independent parameter for survival was investigated.

*Received 8 July 1997*
*Revised 4 December 1997*
*Accepted 6 December 1997*

*Correspondence to:* JMG Bonfrer

### MATERIALS AND METHODS

### Patients

Serum taken before in-house treatment was available from 251 patients, 143 female subjects and 108 male subjects, who were seen at the Netherlands Cancer Institute in the period 1984–94. Their age ranged from 12 to 87 years with a mean age of 50.6 years. The median follow-up time was 67 months with a range of 1–147 months.

The disease stage was determined according to the M.D. Anderson Cancer Centre classification for cutaneous melanoma (Smith, 1976).

Histological classification showed that 46 patients (18%) had superficial spreading, 33 (12%) had nodular melanoma. Amelanotic melanoma was found in 15 (6%) of the cases. Acral lentiginous melanoma was found in 14 (6%). Malignant blue naevi were detected in two and lentigo maligna melanoma in four cases. The pathological diagnosis was not otherwise specified in 138 (55%) patients. Serum from 16 individuals who were treated for various benign skin disorders was also included.

**Table 1** Pretreatment serum concentration of S-100B and neuron-specific enolase

| | n | Median | Min/max | Above 95 percentile[a] |
|---|---|---|---|---|
| *S-100B* | | | | |
| Stage I | 155 | 0.04 | ND–0.16 | 0 |
| Stage II | 15 | 0.05 | ND–0.16 | 0 |
| Stage IIIA | 8 | 0.07 | 0.04–0.19 | 1 (12%) |
| Stage IIIB | 54 | 0.07 | ND–4.69 | 18 (33%) |
| Stage IV | 19 | 1.12 | ND–123.0 | 15 (79%) |
| *NSE* | | | | |
| Stage I | 155 | 5.4 | 1.8–13.0 | 1 (1%) |
| Stage II | 15 | 5.6 | 4.0–8.6 | 0 |
| Stage IIIA | 8 | 5.2 | 3.8–9.4 | 0 |
| Stage IIIB | 53 | 5.4 | 2.1–49.6 | 3 (6%) |
| Stage IV | 19 | 8.0 | 4.7–92.3 | 8 (42%) |

Median values and range of pretreatment serum concentration of S-100B and NSE in patients with malignant melanoma. Results are expressed in μg $l^{-1}$. [a]The 95 percentile used for S-100B was 0.16 μg $l^{-1}$ as found in this study. For NSE 12.5 μg $l^{-1}$ was used as cut-off level (Body et al, 1992).

**Figure 1** Distribution of pretreatment serum S-100B levels in healthy individuals (*n* = 58), patients with benign skin disease (*n* = 16) and cutaneous malignant melanoma, stage I (*n* = 155), stage II (*n* = 15), stage IIIA (*n* = 8), stage IIIB (*n* = 54) and stage IV (*n* = 19). Horizontal lines represent the median values

**Figure 2** Receiver operating characteristic (ROC) curves for S-100B (—) and NSE (- - -) in predicting the presence of extensive disease (stage IIIB and IV) in cutaneous malignant melanoma. The area under the curve was significantly different for S-100B and NSE

To establish a reference range for this new assay we measured the S-100B concentration in 58 normal individuals (41 men and 17 women), who voluntarily donated blood for this occasion. The average age of this group was 62 years, ranging from 40 to 90 years.

### Marker assays

The Sangtec S-100 luminescence immunoassay (LIA) is a two-site immunoluminometric test based on three monoclonal antibodies that specifically bind to the β subunit of the S-100B protein. The assay detects the ββ and αβ dimer in serum (Nyberg et al, 1996).

The standard range is 0.1–20 μg $l^{-1}$ and a lower detection limit of 0.02 μg $l^{-1}$ was established. Day to day variation was found to be 5% at the level of 1.0 μg $l^{-1}$ as well as at 10 μg $l^{-1}$.

The LIA-mat® NSE Prolifigen® assay determines specifically the γ subunit of the enolase enzyme. The reference value has been established at 12.5 μg $l^{-1}$ (Body et al, 1992). The standard range is from 2 to 200 μg $l^{-1}$ and typical day to day CVs were 9% at the lower end and 6% at higher standard levels.

### Statistical methods

The Kruskal–Wallis test is used to compare more than two groups. For the multivariate survival analysis we used the Cox regression model (Cox, 1972). The Spearman coefficient of correlation was used for calculation of correlation between two variables.

Areas under the curve (AUC) for receiver operating characteristic (ROC) curves were compared using the method of Hanley and McNeil (1983).

Constructions of survival curves were carried out according to Kaplan–Meier. Comparisons were performed with the log-rank test.

### RESULTS

The 95 percentile of the group of normal individuals was established at 0.16 μg $l^{-1}$. In 24 of the 58 serum samples we could not

© Cancer Research Campaign 1998

**Figure 3** Survival curves of 251 patients with malignant melanoma according to stage. A total of 155 patients had stage I (—), 23 stage II and IIIA (...), 54 stage IIIB (- - -) and 19 stage IV (□ □ □). Difference between survival in relation to stage is significant ($P < 0.0001$)

**Figure 5** Survival curves of 178 patients with malignant melanoma, limited disease stage I or IIa. A total of 41 patients had S-100B levels > 0.06 μg l$^{-1}$ (- - -), 61 with S-100B between 0.03 and 0.06 (...) and the remaining group of 76 had levels ≤ 0.03 μg l$^{-1}$ (—). The group with levels > 0.06 μg l$^{-1}$ had a worse prognosis ($P = 0.01$)

**Figure 4** Survival curves of 251 patients with malignant melanoma according to the pretreatment serum S-100B level. A positive level (> 0.16 μg l$^{-1}$) was found in 36 patients (- - -). Levels within reference range were found in 215 (—). Difference of survival is highly significant ($P < 0.0001$)

**Figure 6** Survival curves of 55 patients with positive lymph nodes, stage IIIB. Nineteen had elevated pretreatment S-100B levels (> 0.16 μg l$^{-1}$) (- - -) and 36 levels within our reference range (—). Difference between survival time is significant ($P < 0.0001$)

detect any S-100 protein. The highest value found in this group was 0.27 μg l$^{-1}$.

In the 16 patients with benign skin disorder the median S-100 protein concentration could not be calculated because 11 (69%) of the samples had a concentration below the detection limit of 0.02 μg l$^{-1}$. The highest level found was 0.06 μg l$^{-1}$.

The results of the group of patients with malignant melanoma are shown in Table 1. Of the 155 patients with stage I malignant melanoma, 45 (29%) did not have measurable amounts of S-100 protein in pretreatment serum. Fifteen patients (6.0%) had stage II melanoma. In three (20%) patients S-100B was undetectable. Eight patients (3.2%) had stage IIIA disease. In all of these patients S-100B could be detected. Interestingly, 11 of 54 (21.5%) patients with stage IIIB and 1 of 19 with stage IV had undetectable levels of serum S-100B at presentation. A scatterplot of the S-100B concentrations is given in Figure 1.

A Kruskal–Wallis analysis of variance showed a highly significant difference in serum concentration of S-100B protein between stages ($P < 0.00001$). NSE levels showed a less significant difference ($P = 0.0001$).

If levels over 0.16 μg l$^{-1}$ are considered to be elevated, all patients, with the exception of one with stage IIIA (this patient had a level of 0.19 μg l$^{-1}$ and developed lung metastases 11 months later and died 1 year afterwards), with abnormal S-100B protein concentrations had stage IIIB or IV disease. NSE levels over 12.5 μg were also found mainly in the higher stages, but the sensitivity was considerably lower. A ROC curve of S-100B and NSE distinguishing between stages I, II and stage IIIA vs IIIB and IV is shown in Figure 2. The AUC for S-100B was 0.73 ± 0.040 compared with 0.59 ± 0.045 for NSE. This difference is highly significant. At 99% specificity the sensitivity of S-100B is 47% compared with 30% for NSE. Because S-100B was shown to be superior to NSE with respect to sensitivity and specificity we have not included NSE in the survival analyses.

Survival of malignant melanoma patients is related to stage. In the group of patients with limited disease (stage I and II) 5-year

© Cancer Research Campaign 1998

Table 2 *P*-values stepwise Cox regression analysis

| Variable | 0 | 1 | 2 | 3 | 4 | 5 |
|---|---|---|---|---|---|---|
| Stage | 0.0000 | <u>0.0000</u> | <u>0.0000</u> | <u>0.0000</u> | <u>0.0000</u> | <u>0.1397</u> |
| Age | 0.0362 | 0.0548 | 0.0535 | 0.4717 | 0.1831 | 0.1767 |
| PA-diag | 0.0073 | 0.0020 | <u>0.0034</u> | <u>0.0020</u> | <u>0.0247</u> | <u>0.0153</u> |
| Sexe | 0.0824 | 0.6474 | 0.4893 | 0.3248 | 0.3368 | 0.5252 |
| Stage *PA-diag | 0.0000 | 0.0053 | 0.3216 | 0.2878 | 0.0453 | 0.0275 |
| NSE | 0.0643 | 0.3216 | 0.1505 | 0.9717 | 0.8862 | 0.4919 |
| S-100B | 0.0000 | 0.0000 | 0.0000 | <u>0.0000</u> | <u>0.0000</u> | <u>0.7175</u> |
| Stage *S-100B | 0.0000 | 0.0000 | 0.0000 | 0.1495 | 0.0184 | <u>0.0190</u> |
| PA-diag*S-100B | 0.0058 | 0.1310 | 0.0446 | 0.0131 | <u>0.0207</u> | <u>0.0047</u> |

This table shows the results of the Cox regression. In the first block the traditional variables and their interactions were entered. Because of non-linearity of S-100B, S-100B and NSE were divided into five groups: S-100B (µg $l^{-1}$), ≤ 0.02 (60); 0.03–0.04 (60); 0.05–0.06 (46); 0.07–1.0 (38); >1.0 (47); and NSE (µg $l^{-1}$), 1.8–4.3 (49); 4.4–5.0 (47); 5.1–5.8 (58); 5.9–6.7 (45); >6.8 (51). After correction for stage ($P < 0.0001$) and PA-diagnosis ($P = 0.0020$), S-100B was still highly significant ($P < 0.0001$). After entering these three variables there were interactions* between PA-diagnosis and S-100B ($P = 0.0131$) and between stage and S-100B ($P = 0.0184$), which were also entered in the model. Underlined values are entered in the model.

survival was 79% compared with 28% for patients with disseminated disease (Figure 3).

The predictive value of an elevated S-100B level (i.e. > 0.16 µg $l^{-1}$) for survival of patients with malignant melanoma is demonstrated by the Kaplan–Meier curve shown in Figure 4.

The median survival of the group with elevated pretreatment S-100B ($n$ = 36) levels was 7 months, whereas the group of patients with normal S-100B concentration ($n$ = 215) had a median survival of more than 12 years ($P < 0.0001$).

Data on initial tumour thickness were available from 148 patients with stage I, II and IIIA. In a Cox regression model the Breslow thickness of the tumour (Breslow, 1970) was more significantly correlated with survival than S-100B level. After correcting for thickness, S-100B was still a significant factor ($P = 0.02$).

To illustrate the relation of the pretreatment serum S-100B concentration and survival we created three subgroups of comparable size. The first group ($n$ = 41) with a level > 0.06 µg $l^{-1}$, a second ($n$ = 76) with concentrations ≤ 0.03 µg $l^{-1}$ and the remaining patients had a S-100B serum concentration between 0.03 and 0.06 µg $l^{-1}$ (Figure 5). The subgroup with the higher S-100B values had a significantly shorter survival ($P = 0.01$).

Patients with stage IIIB and a pretreatment serum concentration of S-100B below 0.16 µg $l^{-1}$ ($n$ = 36) survived significantly longer than the group with high stage and increased pretreatment S-100B levels ($n$ = 19, Figure 6). The median survival of the latter group was 7 months compared with 34 months for the former group ($P < 0.0001$).

Stage IV with elevated S-100B levels also had a significantly shorter survival than those with levels within the reference range, although all patients died within 2 years.

A stepwise Cox regression analysis was performed to see whether S-100B and NSE provided additional contributions to prognosis using stage, age and tumour histology as variables. After correction for stage ($P < 0.0001$) and histology ($P = 0.003$), S-100B was still a strongly significant factor ($P < 0.0001$). Possible interactions between the parameters were entered in this statistical model (Table 2).

### DISCUSSION

The S-100 protein has been the most practical marker for melanocytic tumours in immunohistochemistry. They may increase the accuracy of melanoma staging and detect metastatic tumour cells not detectable by conventional histology (Cochran et al, 1982).

A promising marker may be a metabolic product of melatonin such as 5-s-cysteinyldopa, and more recently it was reported that the level of soluble intercellular molecule 1 (ICAM-1) is increased in serum of patients with advanced melanoma (Hirai et al, 1997). Both markers seem to have disadvantages, however. Positive 5-s-cd levels were only found in patients with metastatic disease, and sICAM-1 serum levels did not decrease in patients who underwent surgery, which suggests that the serum concentration is not related to tumour stage. NSE is not useful because of the relatively low sensitivity of 25% for extensive disease (Bei Guo et al, 1995).

In this study we measured serum concentrations of S-100B in patients with cutaneous malignant melanoma using the newly developed immunoluminometric assay LIA S-100 (Nyberg et al, 1996). By using an improved labelling system quantification of S-100 protein became possible at the level of 0.02 µg $l^{-1}$.

Von Schoultz et al (1996) did not find levels over 0.20 µg $l^{-1}$ in 81 healthy persons, which is in line with our finding that 95% of our healthy group had levels below 0.16 µg $l^{-1}$. This is not a definitive cut-off limit because of the relatively small number of control subjects, but it is in line with the finding by others, using the less sensitive immunoradiometric assay, that the geometric mean of healthy individuals is below 0.1 µg $l^{-1}$ (Bei Guo et al, 1995; Von Schoultz et al, 1996).

Earlier studies concerning the S-100 serum concentration in patients with malignant melanoma reported lower incidences of elevated S-100 serum levels (Fagnart et al, 1988; Missler and Wiesmann, 1995). The assays in these studies were not based on the same technique, did not use the same antibodies and detection limits of 0.15 µg $l^{-1}$ or higher were found.

Previous studies with the more insensitive immunoradiometric assay (IRMA) using pretreatment serum samples of patients with cutaneous malignant melanoma reported a relation between stage of the disease and the median concentration of S-100B protein, although an adequate assessment of the sensitivity of the S-100B was not possible (Von Schoultz et al, 1996; Henze et al, 1997). Bei Guo et al (1995) found positive S-100B values in 13% of stage IIIB patients, contrary to 33% in this study.

In this study we confirmed a significant correlation between stage and disease. Levels of S-100 over 0.16 µg $l^{-1}$ indicate extensive

© Cancer Research Campaign 1998

disease, and stage I and IIA patients were inclined to have serum levels below 0.16 μg $l^{-1}$.

The finding that 85% of patients with metastatic disease had elevated S-100B concentrations is notable. This result suggests that S-100 has at least an equal sensitivity for malignant melanoma as established tumour markers in other situations, e.g. CA 125 in ovarian carcinoma (Tuxen et al, 1995).

The prognosis for patients with malignant melanoma is determined by the stage of the disease. The M.D. Anderson classification for staging does not include the tumour thickness or level of invasion, two features known to affect prognosis. Especially in cases of limited disease, stage I and II, tumour thickness is considered to be the single most important prognostic factor (Balch et al, 1992). The finding that S-100B was a significant independent prognostic factor after correction for tumour thickness has not been reported before. The fact that the expression of the β subunit of S-100 protein in malignant melanoma is associated with vertical growth and invasiveness may be responsible for this unique result (Cho et al, 1990).

Pretreatment serum levels of S-100B protein can identify patients with a high risk of early death in the group of patients with stage IIIB and stage IV disease. This may have implications in the treatment to be chosen.

The classification used for staging does not allow us to differentiate this group of patients according to the number of lymph nodes that were found positive during surgical exploratory procedures. However, ongoing trials to study the effect of the search for sentinel nodes (Day and Lew, 1985) may facilitate the validation of S-100B protein as a marker for extensive disease.

The most important application of tumour markers has been as an indicator for recurrence during follow-up. It has been shown that serial determinations of the serum S-100B concentration may be of help in the assessment of response to treatment (Bei Guo et al, 1995; Missler and Wiesmann, 1995). In a recent study it was found that in patients with high risk of recurrence and an elevated level of S-100B at any moment after initial treatment had a significantly shorter time to relapse than the group with no elevation (Miliotes et al, 1996). The relatively low incidence (48%) of elevated levels in this publication might be due to the insensitivity of the assay used. It is important to investigate the use of the LIA S-100 assay to detect recurrence at an early stage and so enhance the effectiveness of repeated treatment.

The availability of this sensitive assay for the detection of S-100B protein in serum will improve the utility of the protein as a marker for early recurrence as successive increases of the marker at low concentrations may indicate relapse.

### REFERENCES

Balch CM, Soong SJ, Shaw HM, Urist MM, McCarthy WH (1992) An analysis of prognostic factors in 8500 patients with cutaneous melanoma. In *Cutaneous Melanoma*, Balch CM and Milton GW (eds), pp. 165–187. Lippincott: Philadelphia

Baudier J, Glasser N, Gerard D (1986) Ions binding to S-100 proteins. *J Biol Chem* **261**: 8192–8203

Bei Guo H, Stoffel-Wagner B, Bierwirth T, Mezger J, Klingmüller D (1995) Clinical significance of serum S-100 in metastatic malignant melanoma. *Eur J Cancer* **31A**: 1898–1902

Body JJ, Paesmans M, Sculier JP, Dabouis G, Bureau G, Libert P, Berchier MC, Raymakers N and Klastersky J (1992) Monoclonal immunoradiometric assay and polyclonal radio immunoassay compared for measuring neuron-specific-enolase in patients with lung cancer. *Clin Chem* **38**: 748–751

Breslow A (1970) Thickness, cross-sectional areas and depth of invasion in the prognosis of cutaneous melanoma. *Ann Surg* **172**: 902–908

Cho KH, Hashimoto K, Taniguchi Y, Pietruk T, Zarbo RJ and An T (1990) Immunohistochemical study of melanocytic naevus and malignant melanoma with monoclonal antibodies against S-100 subunits. *Cancer* **66**: 765–771

Cochran AJ, Wen DR, Herschman HR, Gaynor RB (1982) Detection of S-100 protein as an aid to the identification of melanocytic tumours. *Int J Cancer* **30**: 295–297

Cochran AJ, Lu HF, Li PX, Saxton R, Wen DR (1993) S-100 protein remains a practical marker for melanocytic and other tumours. *Melanoma Res* **3**: 325–330

Cox DR (1972) Regression models life tables. *J R Stat Soc* **34**: 187–220

Day CL and Lew RA (1985) Malignant melanoma prognostic factors: elective lymph node dissection. *J Dermatol Surg Oncol* **11**: 233–239

Fagnart OC, Sindic CJ, Laterre C (1988) Particle counting immunoassay of S-100 protein in serum. Possible relevance in tumours and ischemic disorders of the central nervous system. *Clin Chem* **34**: 1387–1391

Gaynor R, Irie R, Morton DL and Herschman HR (1980) S-100 protein in cultured human malignant melanomas. *Nature* **286**: 400–401

Hanley JA and McNeil BJ (1983) A method of comparing the areas under receiving operating characteristics curves derived from the same cases. *Radiology* **148**: 839–843

Henze G, Dummer R, Joller-Jemelka HI, Böni R and Burg G (1997) Serum S100 – a marker for disease monitoring in metastatic melanoma. *Dermatology* **194**: 208–212

Hirai S, Kageshita T, Kimura T, Tsujisaki M, Imai K, Wakamatsu K, Ito S and Ono T (1997) Serum levels of sICAM-1 and 5-S-cysteinyldopa as markers of melanoma progression. *Melanoma Res* **7**: 58–62

Miliotes G, Lyman GH, Cruse CW, Puleo C, Albertini J, Rapaport D, Glass F, Fenske N, Soriano T, Cuny C, Van Voorhis N and Reintgen D (1996) Evaluation of new putative tumour markers for melanoma. *Ann Surg Oncol* **3**: 558–563

Missler U and Wiesmann M (1995) Measurement of S-100 protein in human blood and cerebrospinal fluid: analytical method and preliminary clinical results. *Eur J Clin Chem Clin Biochem* **33**: 743–748

Nyberg L, Kroon R, Ullén A, Brundell J, Haglid KG and Stigbrand T (1996) Sangtec® 100 LIA a new sensitive monoclonal assay for measuring protein S-100 in patients with malignant melanoma. In *Final Program and Abstract Book of the XXIV Meeting of the ISOBM*, The Interdependence of Tumour Biology and Clinical Oncology p. 108. San Diego, CA

Schäfer BW and Heizmann CW (1996) The S100 family of EF-hand calcium-binding proteins: functions and pathology. *TIBS* **21**: 134–140

Smith JL (1976) Histopathology and biological behavior of melanoma. In *Neoplasms of the Skin and Malignant Melanomas*. Year Book Medical Publishers: Chicago

Tuxen MK, Sölétormos G and Dombernowsky P (1995) Tumor markers in the management of patients with ovarian cancer. *Cancer Treat Rev* **21**: 215–245

von Schoultz E, Hansson LO, Djureen E, Hansson J, Kärnell R, Nilsson B, Stigbrand T and Ringborg U (1996) Prognostic value of serum analyses of S-100β protein in malignant melanoma. *Melanoma Res* **6**: 133–137

Wibe E, Paus E and Aamdal S (1990) Neuron-specific enolase in serum of patients with malignant melanoma. *Cancer Lett* **52**: 29–31

*© Cancer Research Campaign 1998*